# Single-Cell Analysis Highlights Pivotal Role of Eosinophil–Basophil Mast Cell Progenitor-Related Mechanism in Primary Immune Thrombocytopenia

**DOI:** 10.3390/ijms27083535

**Published:** 2026-04-15

**Authors:** Mei Xie, Haimei Deng, Fangjie Liu, Wei Xiao, Xiaojun Xu, Rongli Xie, Tiantian Sun

**Affiliations:** Department of Hematology, The Seventh Affiliated Hospital, Sun Yat-sen University, Shenzhen 518107, China; xiemei@sysush.com (M.X.); denghaimei@sysush.com (H.D.); liufangjie@sysush.com (F.L.); xiaowei@sysush.com (W.X.); xuxiaojun@sysush.com (X.X.)

**Keywords:** immune thrombocytopenia, single cell sequencing, pre-B cell populations, eosinophil-basophil mast-cell progenitors, LGALS9-CD44

## Abstract

Immune thrombocytopenia (ITP) is an autoimmune disease. Megakaryocyte dysfunction caused by autoimmune response can lead to thrombocytopenia, and the underlying mechanism is still unclear. Single-cell sequencing analysis revealed the heterogeneity of CD34 + HSPCs in bone marrow between ITP patients and healthy groups. Pre-B cell population 1 (pre-B1) showed a significantly lower percentage contribution in ITP groups, and the underlying mechanism involves cell cycle-, cell apoptosis- and cell death-related pathways. The number of eosinophil–basophil mast cell progenitors (EBMPs) is significantly increased in ITP patients and the DEGs of the EBMPs in ITP patients were significantly enriched in immune-related pathways. Further, immunofluorescent staining and Western blot assay highlight C-X-C Motif Chemokine Ligand 8 (CXCL8) and Interferon Regulatory Factor 1 (IRF1) expression were significantly increased in the EBMPs of ITP patients. Furthermore, cell–cell communication analysis identified an impaired LGALS9-CD44 axis between EBMP cells and MkP1 cells in ITP patients, suggesting that targeting the LGALS9-CD44 interaction might hold promise as a therapeutic approach for ITP. Our observations indicate that ITP patients exhibit an elevated proportion of EBMP cells alongside a reduced proportion of pre-B1 cells. CXCL8 and IRF1 are potentially associated with EBMP cell dysfunction and the ITP disease process. Furthermore, the diminished LGALS9-CD44 axis between EBMP and MkP1 cells may contribute to ITP progression, suggesting a direction for future therapeutic investigation.

## 1. Introduction

Primary immune thrombocytopenia (ITP) is an autoimmune disease characterized mainly by a decrease in platelet count [1]. Its clinical manifestations are mostly skin and mucosal bleeding, making it the most common bleeding autoimmune disease in clinical practice [2]. The pathogenesis of ITP is currently not clear, with most scholars believing that impaired maturation of megakaryocytes, abnormal antigen-specific autoantibodies, and T cell-mediated platelet destruction are important mechanisms in the development of ITP [3,4]. The standard first-line treatment for ITP remains glucocorticoids, which mainly target the immune system to reduce the attack on platelets and increase the platelet count in patients [5,6]. Despite new treatment methods emerging in recent years, around 30–40% of patients still do not respond to treatment or experience relapses in the short term, progressing to chronic or refractory ITP [7]. Therefore, further exploration of the pathogenesis of ITP and the search for new ITP-specific treatment methods are still urgent issues that need to be addressed.

Hematopoietic stem and progenitor cells (HSPCs) are a type of multipotent stem cell found in the bone marrow, possessing the ability to self-renew and differentiate into various types of blood cells [8]. HSPCs are the foundation of the blood and immune systems, capable of differentiating into red blood cells, white blood cells, and platelets, among other types of blood cells [9]. Subpopulations of HSPCs have the capacity to regulate immune responses, influencing inflammation and immune tolerance. Research on HSPCs is crucial for understanding the pathogenesis of blood diseases, developing new therapeutic strategies, and improving the treatment outcomes of blood disorders. For instance, in the disease ITP (immune thrombocytopenia), the behavior and function of HSPCs may undergo changes that affect platelet survival and immune regulation.

Platelet production is a complex biological process, including hematopoietic stem cell-directed differentiation, proliferation, and terminal differentiation of megakaryocyte progenitor cells (MkPs), megakaryocyte maturation, and platelet release [10,11]. Megakaryocytes differentiate from hematopoietic stem cells to maturity, with mature megakaryocytes shedding and decomposing into multiple reticulated platelets, released into the bloodstream as mature platelets to participate in the body’s coagulation and inflammatory reactions [12,13]. Platelet destruction activates bone marrow hematopoiesis, and hematopoietic stem/progenitor cells (HSPCs) themselves may also be targets of autoimmune attacks. Bone marrow immune tolerance defects are one of the main reasons for megakaryocyte maturation disorders and decreased platelet production in ITP [14]. Studies have shown that reduced expression of tumor necrosis factor-related apoptosis-inducing ligand (TRAIL) in ITP can lead to megakaryocyte maturation disorders, with bone marrow megakaryocytes undergoing apoptosis influenced by CD8+ T cells, resulting in decreased platelet production [15,16]. The role played by HSPCs in this process is currently not clear.

Research on the mechanism of thrombocytopenia has traditionally focused on megakaryocyte development and abnormal platelet production, with little attention given to hematopoietic stem/progenitor cells [10,17]. Previous studies have suggested that the heterogeneity of mature bone marrow megakaryocyte progenitor cells and pre-B cells may serve as potential targets for ITP treatment [18]. Moreover, intercellular communication between different subpopulations of HSPCs may play a crucial role in inducing defective megakaryocyte generation. In this study, we will analyze data from single-cell transcriptomics of bone marrow hematopoietic stem/progenitor cells, perform intercellular interaction analysis, and study clinical samples to identify the key mechanisms of immune abnormalities underlying the pathogenesis of ITP.

## 2. Results

### 2.1. Single-Cell Sequencing Analysis Revealed the Heterogeneity of CD34 + HSPCs in the BM of ITP Versus Healthy Patients

**Figure 1:**
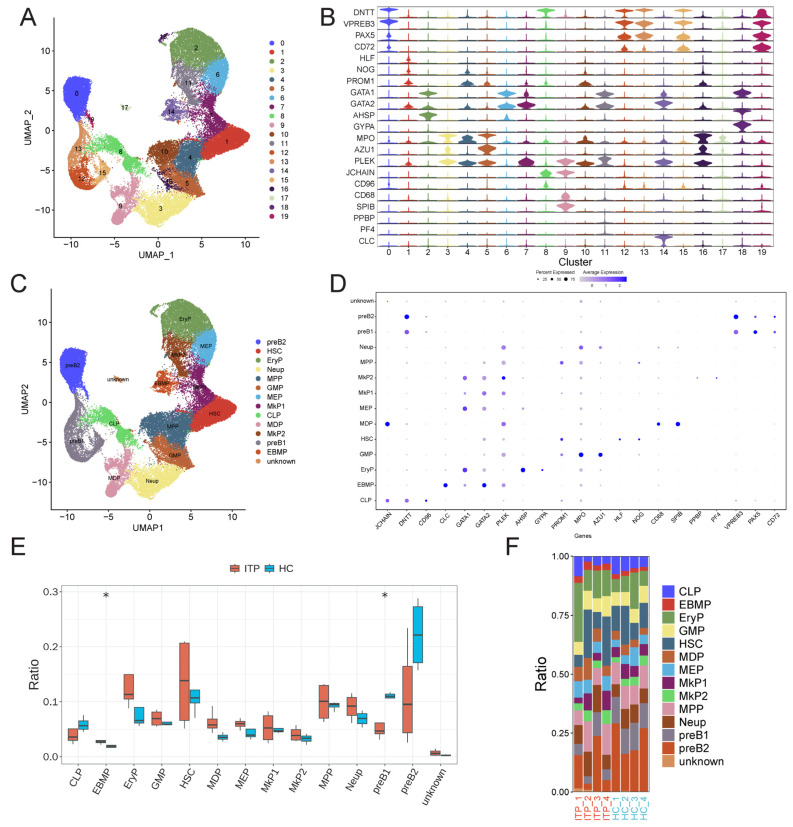
Identification of hematopoietic stem and progenitor cells (HSPCs) in bone marrow samples from ITP patients and healthy controls (HCs). (**A**) UMAP visualization of cell clusters derived from bone marrow single-cell transcriptomic profiling. (**B**) Violin plots illustrating the expression patterns of signature genes across distinct cell clusters. (**C**) UMAP plot colored by annotated cell types. Each dot represents a single cell. CLPs: common lymphoid progenitors; EBMPs: eosinophil–basophil mast cell progenitors; EryPs: erythroid progenitors; GMPs: granulocyte macrophage progenitors; HSCs: hematopoietic stem cells; MDPs: monocyte-dendritic-cell progenitors; MEPs: megakaryocyte-erythroid progenitors; MkP1s: megakaryocytic progenitors 1; MkP2s: megakaryocytic progenitors 2; MPPs: multipotent progenitors; Neups: neutrophil progenitors; pre-B1: pre-B cell population 1; pre-B2: pre-B cell population 2. (**D**) Bubble plot showing the expression of cell-type-specific marker genes. Color intensity represents the average expression level, and dot size indicates the percentage of cells expressing the corresponding gene. (**E**) Bar plots comparing the proportion of each annotated cell type between the ITP and HC groups. Bar plots showing the percentage contribution of each cell type across all analyzed samples. (**F**) Stacked bar plot showing the cell composition ratio of each cluster in ITP and healthy control (HC) groups. * *p* < 0.05.

To investigate the hematopoietic transcriptional landscape and determine the alterations in ITP, we downloaded scRNA-seq of BM CD34 + HSPCs from four ITP patients and four healthy donors from the GEO dataset (https://www.ncbi.nlm.nih.gov/geo/query/acc.cgi?acc=GSE196676 (accessed on 30 March 2026)). We first assigned cell-type labels to cells within the scRNA-seq dataset using a cluster-based approach. All 56,312 cells were acquired, including 27,805 cells in the HC group and 28,507 cells in the ITP group. We annotated clusters using differentially expressed genes and visualized them with Uniform Manifold Approximation and Projection (UMAP). This analysis revealed 19 cell clusters of HSPCs in ITP and HC patient bone marrow (Figure 1A). All 19 clusters were evenly distributed between the ITP and HC groups (Figure S1A,B). The violin plot displays the specificity of marker genes of 19 cell clusters in the ITP and HC patient bone marrow samples (Figure 1B). Next, all 19 clusters were classified into 13 cell types with distinct gene expression patterns (Figure 1D and Figure S2B), including common lymphoid progenitors (CLPs), eosinophil–basophil mast cell progenitors (EBMPs), erythroid progenitors (EryPs), granulocyte macrophage progenitors (GMPs), hematopoietic stem cells (HSCs), monocyte-dendritic-cell progenitors (MDPs), megakaryocyte erythroid progenitors (MEPs), megakaryocytic progenitors (MkP1s), megakaryocytic progenitors 2 (MkP2), multipotent progenitors (MPPs), neutrophil progenitors (Neups), pre-B cell population 1 (pre-B1), and pre-B cell population 2 (pre-B2) (Figure 1C). All 13 cell types were evenly distributed between the ITP and HC groups (Figure S2A).

To explore the differences in cell composition between the ITP disease group and the HC control group, and further investigate the core cell populations related to ITP onset, we analyzed the proportions of different cell populations in the ITP and HC groups. The results showed that the proportion of EBMP cells significantly increased in the disease group, while the proportion of pre-B1 cells significantly decreased in the ITP groups (Figure 1E). Moreover, the proportion distribution plot shows that pre-B1 cells accounted for the majority in both groups (Figure 1F). The above results suggest that the EBMP and pre-B1 cell populations may play a key role in the pathogenesis of ITP.

### 2.2. Differentially Expressed Genes in Pre-B1 Were Enriched in Cell Cycle-, Cell Apoptosis- and Cell Death-Related Pathways

**Figure 2:**
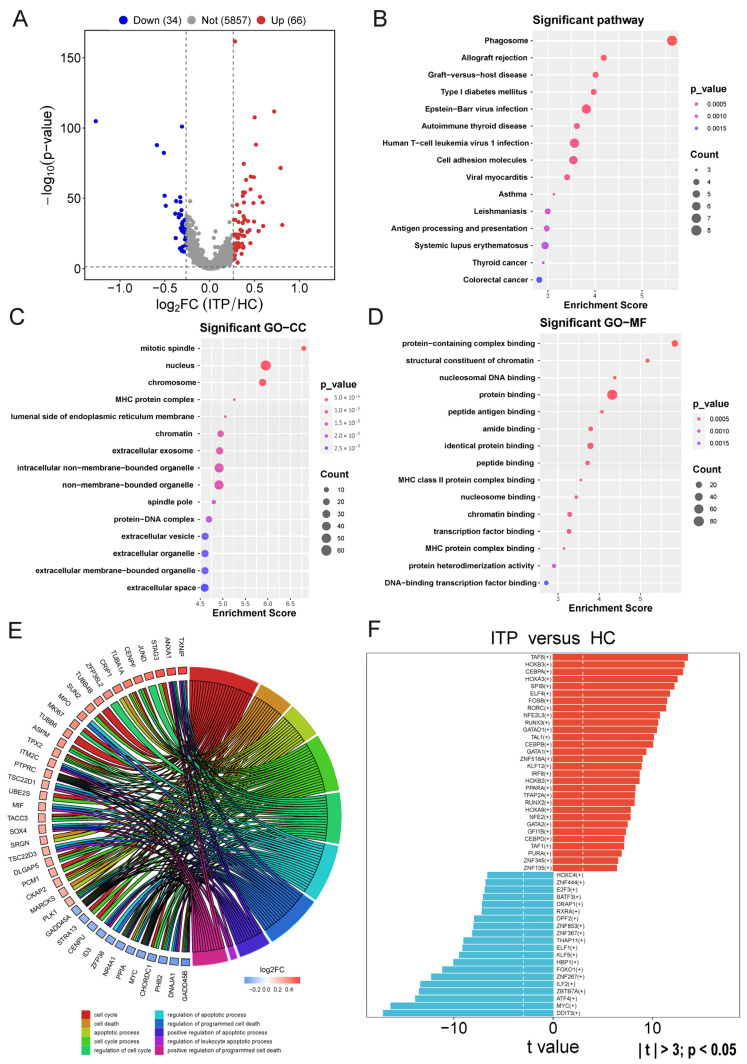
Differentially expressed genes and functional enrichment analysis in pre-B1 cell clusters. (**A**) Volcano plot showing differentially expressed genes (DEGs) in pre-B1 cells between ITP patients and healthy controls (HCs). Red dots indicate upregulated genes and blue dots indicate downregulated genes. (**B**) Top 15 enriched KEGG pathways of DEGs in pre-B1 cells of ITP BM. (**C**) Top 15 enriched cellular component terms of DEGs in pre-B1 cells of ITP BM. (**D**) Pre-B1-cell-specific significant GO-MF enrichment in ITP patients. (**E**) The pre-B1-cell-specific significant GO-BP function enrichment and differentially expressed genes in ITP patients. (**F**) SCENIC analysis identifying key transcription factor regulons associated with DEGs in pre-B1 cells in ITP patients.

The pre-B1 cell is an early stage of B cell development, closely related to the maturation and function of the immune system. The reduction in pre-B1 cells in ITP may be related to the inhibition of B cell development and immune dysregulation. Based on the distribution of cell types, we selected pre-B1 cells as the core cell clusters for subpopulation analysis in ITP BM. In order to explore the underlying mechanisms of the core cell clusters in the pathogenesis of ITP, we used the FindMarkers function in the Seurat package (version 5.4.0) to perform inter-group differential expression analysis of core cell clusters between the ITP and HC groups. The screening criteria were set as an |FC| > 1.2 and a *p* value < 0.05. As shown in Figure 2A, the volcano plot displays a total of 100 differentially expressed genes in pre-B1 cells between the HC and ITP groups, including 66 significantly upregulated genes and 34 significantly downregulated genes. Next, functional enrichment analysis will be conducted on the differentially expressed genes in pre-B1 cells between the HC and ITP groups. The top 15 enriched pathways are shown in Figure 2B, including those for autoimmune thyroid disease, cell adhesion molecules, antigen processing and presentation, etc. The functions of the MHC protein complex, extracellular vesicles, and extracellular membrane-bounded organelle were enriched in the GO CC term of ITP patients (Figure 2C). The GO MF term of protein binding, peptide binding, MHC class II protein complex binding, and transcription factor binding was enriched in the ITP group (Figure 2D). Furthermore, the expression of TXNIP, ANXA1, STAG3, and JUND were upregulated in the pre-B1 cells of ITP patients, and the DEGs of pre-B1 cells in ITP patients were significantly enriched in cell cycle, cell death, and apoptotic processes and regulation of the cell cycle, regulation of apoptotic process, regulation of programmed cell death, and positive regulation of programmed cell death (Figure 2E). HOXB3 has been implicated in the regulation of hematopoietic development and immune responses, making it a candidate of interest in the context of ITP pathogenesis. Moreover, we used the SCENIC package to explore the over-activated transcription factor HOXB3 in the ITP group compared to the HC group within the pre-B1 cell subpopulation (Figure 2F). Thus, the above results indicated that the differential genes in the pre-B1 cells of ITP patients were related to the cell cycle, cell apoptosis and cell death pathways.

### 2.3. The Increase in EBMP Cells Was Identified by Marked CLC Genes in the ITP Sample

**Figure 3:**
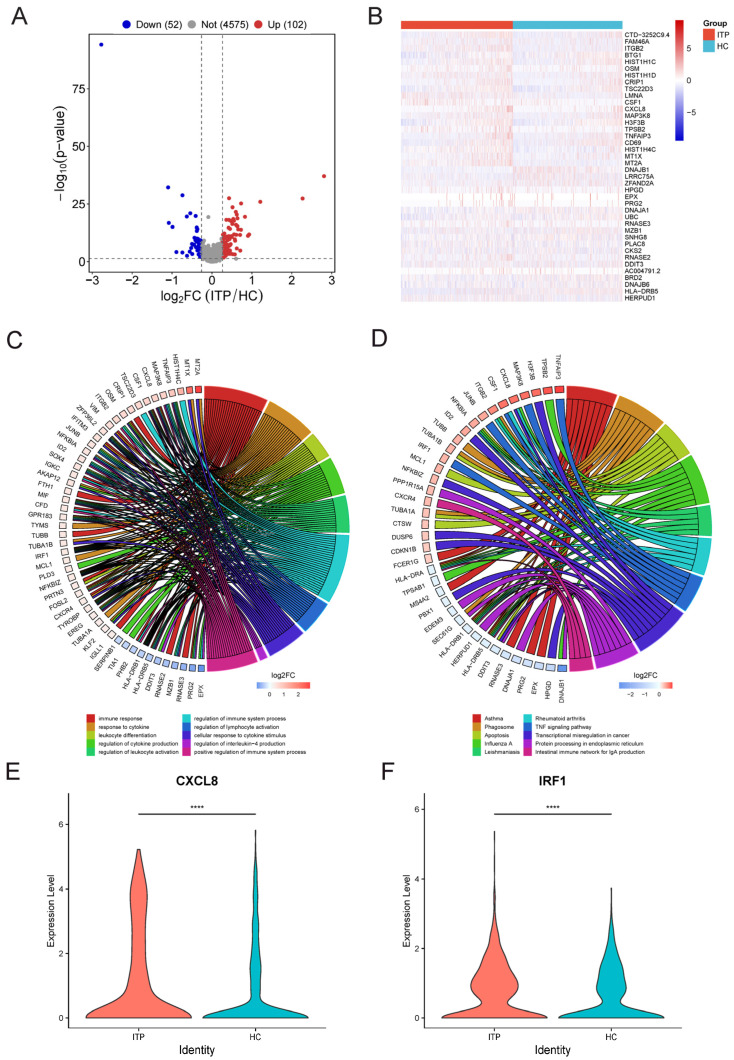
Differentially expressed genes and functional enrichment analysis in EBMP cell clusters. (**A**) Volcano plot showing differentially expressed genes (DEGs) in EBMP cells between healthy controls (HCs) and ITP patients. Red dots indicate upregulated genes, and blue dots indicate downregulated genes. (**B**) Heatmap of the top 20 differentially expressed genes in EBMP cells between the HC and ITP groups. Red indicates upregulated genes, and blue indicates downregulated genes. (**C**) Top 15 significantly enriched KEGG pathways based on DEGs in EBMP cells from ITP bone marrow. (**D**) Significantly enriched GO biological process (BP) terms and corresponding DEGs specific to EBMP cells in ITP. (**E**,**F**) Expression levels of *C-X-C Motif Chemokine Ligand 8* (*CXCL8*) and *Interferon Regulatory Factor 1* (*IRF1*) in EBMP cells between ITP and HC groups. **** *p* < 0.001.

Next, we focus on another core cell cluster, which is EBMP. EBMP cells are a key cell population involved in immune responses and inflammation processes, playing a role in regulating immune responses and inflammation. The pathological mechanism of ITP involves platelet destruction caused by autoimmunity and dysfunction of megakaryocytes in the bone marrow. The increase in EBMP cells may be associated with enhanced inflammation and immune responses in ITP. Firstly, on the basis of the expression of four well-known genes (*CLC*, *GATA1*, *GATA2*, and *PLEK*), we identified the EMBP cell type of the ITP BM. As shown in Figure 3A, the UMAP map shows the EMBP cell cluster of the ITP BM. Previous analysis indicated that the proportion of EBMP cells in ITP patients was significantly increased compared with the HC groups. Further, we collected bone marrow samples from 15 ITP cases and 5 healthy control cases and used immunofluorescence to validate the expression levels of the marker gene CLC in the EBMP cell cluster in the ITP group and control group samples. The clinical characteristics of the active ITP patients are shown in Table 1. As shown in Figure 2B, compared with the HC group, the levels of the marker gene CLC in the EBMP cells of the ITP group were significantly increased. Thus, the increase in EBMP cells was identified by marked CLC genes in the ITP samples.

### 2.4. Differentially Expressed Genes in EBMP Were Enriched in Immune and Inflammatory Response-Related Pathways

Based on the above analysis, we speculate that EBMP plays an important role in ITP. In order to explore the mechanism of core cell EBMP in the pathogenesis of ITP, we used FindMarkers in the Seurat package to analyze the differential expression of core cells between the ITP and HC groups. The screening criteria were an |FC| > 1.2 and a *p*-value < 0.05. As shown in Figure 3A, the volcano plot displays a total of 154 differentially expressed genes in EBMP cells between the HC and ITP groups, including 102 significantly upregulated genes and 52 significantly downregulated genes. The heatmap shows the top 20 DEGs in the EBMP cells of the ITP and HC groups (Figure 3B).

**Figure 4:**
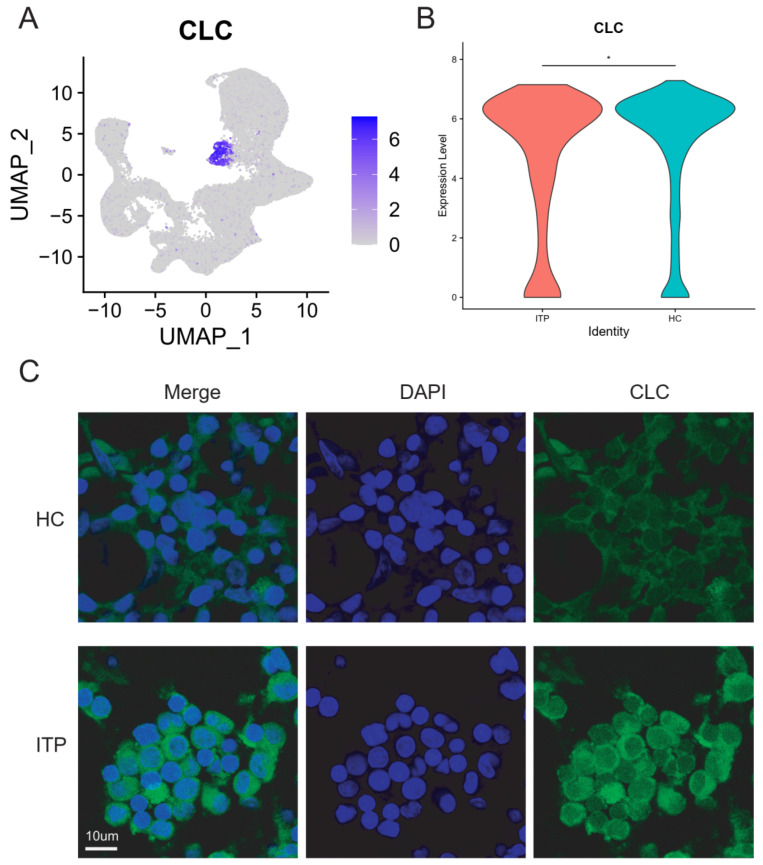
Identification of EBMP cell clusters by immunofluorescence staining in ITP and healthy bone marrow samples. (**A**) UMAP visualization of CLC expression in bone marrow cells from ITP patients and healthy controls (HCs). (**B**) Comparison of CLC expression levels in bone marrow samples between the ITP and HC groups. (**C**) Immunofluorescence staining validating the expression of CLC in EBMP cell clusters from ITP and HC bone marrow samples. Scale bar: 10 μm. * *p* < 0.05.

Next, functional enrichment analysis will be conducted on the differentially expressed genes in EBMP cells between the HC and ITP groups. The top 15 enriched pathways are shown in Figure 4C; the KEGG TNF signaling pathway, intestinal immune network for IgA production, protein processing in endoplasmic reticulum, and apoptosis were significantly enriched. Furthermore, the DEGs of the EBMPs in the ITP group were significantly enriched in immune-related pathways, including immune response, response to cytokine, leukocyte differentiation, regulation of cytokine production, regulation of immune system process, and cellular response to cytokine stimulus (Figure 3D). Immune inflammation-related gene *C-X-C Motif Chemokine Ligand 8* (*CXCL8*) and *Interferon Regulatory Factor 1* (*IRF1*) expression was significantly increased in the EBMP cells of ITP patients (Figure 3E,F). Moreover, we used the SCENIC package to explore the over-activated transcription factor FosL1 in the ITP group compared to the HC group within the EBMP cell subpopulation (Supplementary Figure S3). Thus, the above results indicated that the differential genes in the EBMP cells of the ITP group were related to immune system processes and inflammatory response.

### 2.5. High Expression of CXCL8 and IRF1 Is Related to the Immune Dysregulation of EBMP Cells in Patients with ITP

**Figure 5:**
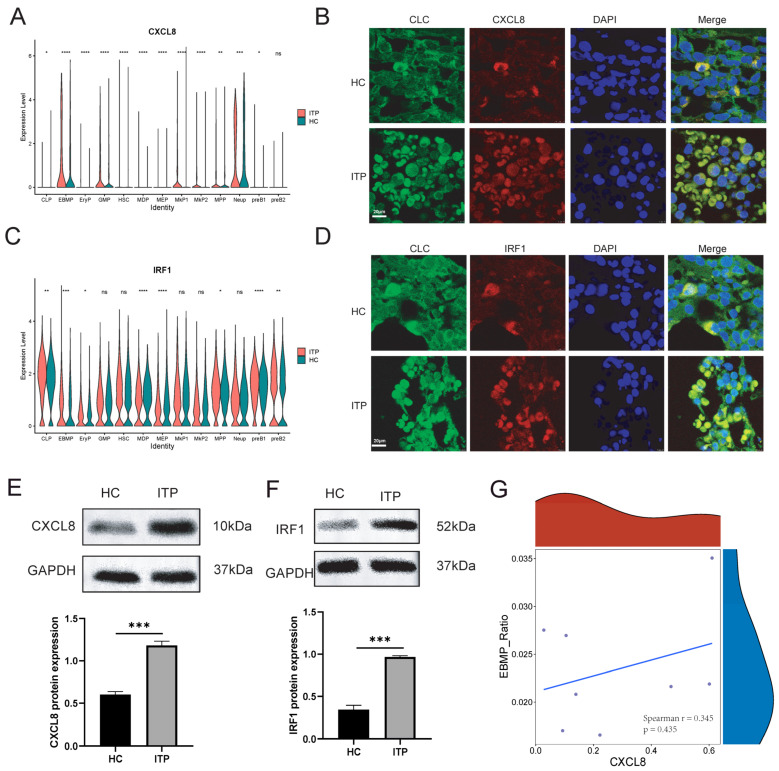
Validation of CXCL8 and IRF1 expression in EBMP cell clusters from ITP and HC bone marrow samples. (**A**) Violin plots showing mRNA expression levels of CXCL8 across distinct hematopoietic cell subpopulations in ITP and HC groups. (**B**) Double immunofluorescence staining of CLC (EBMP marker, green) and CXCL8 (red) in bone marrow sections from HC and ITP patients. Nuclei were counterstained with DAPI (blue). (**C**) Violin plots showing mRNA expression levels of IRF1 across distinct hematopoietic cell subpopulations in ITP and HC groups. (**D**) Double immunofluorescence staining of CLC (EBMP marker, green) and IRF1 (red) in bone marrow sections from HC and ITP patients. Nuclei were counterstained with DAPI (blue). (**E**) Western blot analysis showing significantly higher CXCL8 protein expression in the ITP group than in the HC group. (**F**) Western blot analysis showing significantly higher IRF1 protein expression in the ITP group than in the HC group. (**G**) Correlation analysis between CXCL8 expression levels and EBMP cell counts in ITP patients (Spearman r = 0.345, *p* = 0.435). Scale bar: 10 μm. The red and blue areas in the marginal distributions represent the density distributions of the EBMP_Ratio (red, y-axis) and CXCL8 (blue, x-axis) variables, respectively. Note: ns = not significant, indicating no statistically significant difference; * = *p* < 0.05, ** = *p* < 0.01, *** = *p* < 0.001, **** = *p* < 0.0001, indicating statistically significant differences compared with the control group.

To further explore the correlation between ITP and the expression of immune and inflammation-related proteins, the potential molecular mechanisms of ITP were elucidated through analyzing the expression of the CXCL8 and IRF1 proteins in different hematopoietic cell subpopulations. CXCL8 expression was significantly increased in EBMP, CLP, EryP, GMP, HSC, MDP, MEP, MkP1, MkP2, MPP, Neup, and pre-B1 cells of ITP (Figure 5A). Further confirmation using immunofluorescence staining demonstrated that CXCL8 expression was increased in the EBMP cells of ITP patients compared with the HC group (Figure 5B). Next, IRF1 expression was significantly increased in CLP, EBMP, EryP, MDP, MEP, MPP, pre-B1, and pre-B2 single cells of ITP patients (Figure 5C). Immunofluorescence staining further confirmed IRF1 expression was increased in the EBMP cells of the ITP group compared with HC group (Figure 5D). Additionally, Western blot analysis suggested that CXCL8 protein expression was higher in the ITP group than the HC group (Figure 5E). The expression of IRF proteins was also upregulated in the ITP group (Figure 5F). Although the correlation analysis revealed a positive trend between CXCL8 expression and EBMP cell proportion in ITP patients, this association did not reach statistical significance (Figure 5G, *p* > 0.05). These results indicate that the high expression of CXCL8 and IRF1 in patients with ITP is related to the immune dysregulation of EBMP cells.

### 2.6. EBMP-MkP1 Cell Communication Networks Were Identified in ITP Patients

**Figure 6:**
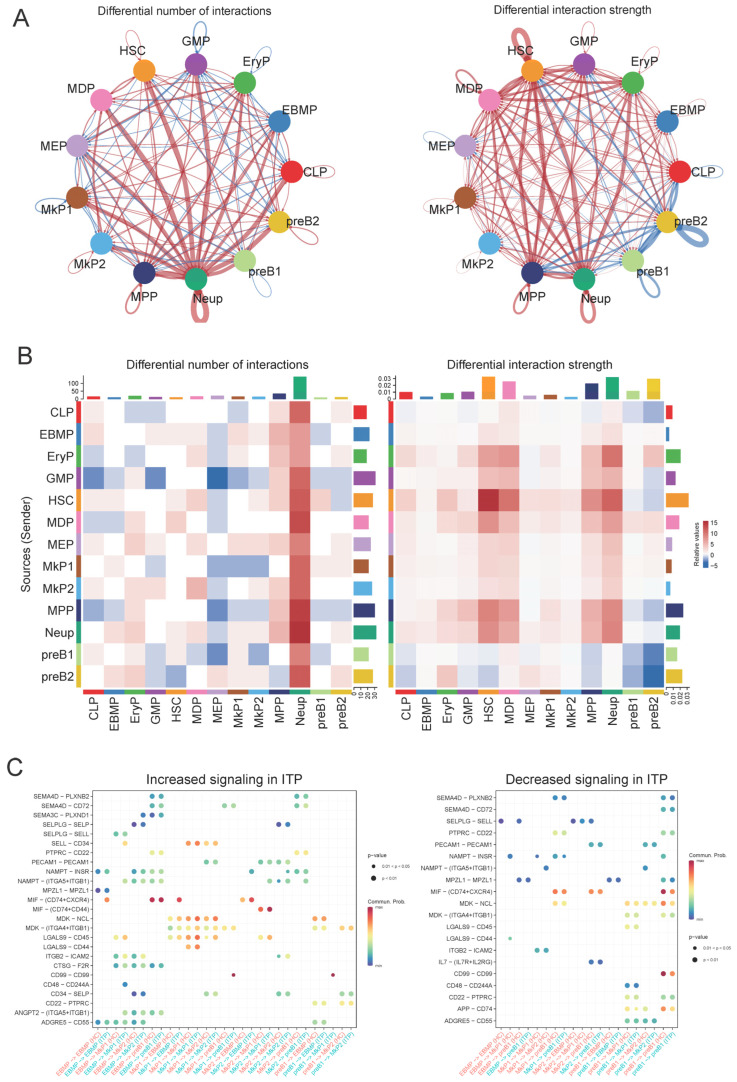
Global cell–cell communication patterns and associated signaling pathways in ITP. (**A**) Network plot illustrating cell–cell interactions; line width corresponds to the number and strength of intercellular communications. (**B**) Incoming and outgoing signaling patterns of each cell phenotype in ITP bone marrow, highlighting dominant signaling pathways. (**C**) Communication strength of all significant signaling pathways mediating cell–cell crosstalk in ITP.

As previously mentioned, there is no understanding of how HSPC phenotypes occur in ITP. We hypothesize that intercellular communication among distinct HSPC phenotypes may accelerate this process. Therefore, the R package “CellChat” (version 1.1.3) was used to investigate differences between various HSPC phenotypes. The number of cell–cell communications and the interaction strength is shown for 13 HSPC phenotypes (Figure 6A). The incoming/outgoing communication patterns of HSPC populations are also shown (Figure 6B). The unique absence of the LGALS9-CD44 axis in ITP patients suggests a potential loss of regulatory control over EBMP and MkP1 cell interactions, which could contribute to the dysregulated immune response characteristic of ITP (Figure 6C).

### 2.7. Validation of Key Ligand–Receptor Pairs by Dual IHC Staining in ITP Samples

**Figure 7:**
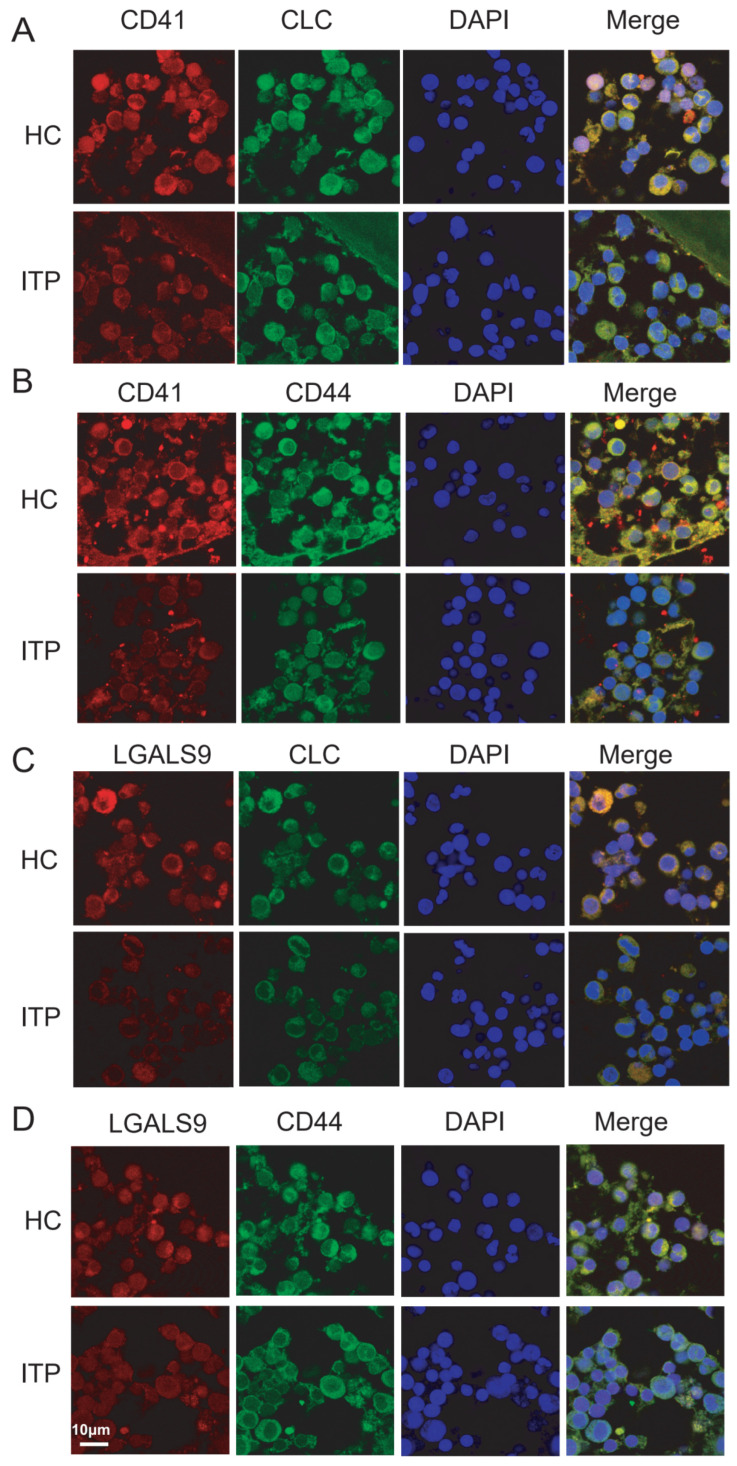
Co-localization of ligand–receptor pairs in bone marrow by double immunofluorescence. (**A**) Double immunofluorescence staining of CD41 and CLC. (**B**) Double immunofluorescence staining of CD41 and CD44; (**C**) Double immunofluorescence staining of LGALS9 and CLC; (**D**) Double immunofluorescence staining of LGALS9 and CD44. Representative images show co-localization of CD41 (red) and CLC (green) in bone marrow sections. Merged signals (yellow) indicate cells positive for both markers, implying potential intercellular interactions or a distinct cell state. Scale bar: 10 μm. No quantitative statistical analysis was performed.

The ligand gene *LGALS9* on the EBMP cell cluster in the disease group and control group samples co-localizes with the receptor gene *CD44* on the MkP1 cell cluster. Firstly, there is a unique LGALS9-CD44 axis between the EBMP cell population (CLC) and the MkP1 cell population (CD41) in the normal group, which is absent between the EBMP and MkP1 cell populations in the disease group (Supplementary Figure S4). CD41 is typically associated with megakaryocytic progenitors (MkP1s) and CLC with eosinophil–basophil mast cell progenitors (EBMPs). Next, double immunofluorescence staining was used to observe the co-expression of CD41 and CLC, CD41 and CD44, LGALS9 and CLC, and LGALS9 and CD44 in bone marrow samples from the ITP and HC groups. Compared with the ITP group, the staining intensity of CD41 + CLC in the normal group was significantly increased (Figure 7A). Similarly, the staining intensity of CD41 + CD44 in the normal group was significantly increased (Figure 7B); whereas, the staining intensity of LGALS9 + CLC in the normal group was strong (Figure 7C), and the intensity of LGALS9 + CD44 in the ITP group was significantly lower than that in the normal group (Figure 7D).

## 3. Discussion

ITP is the most common hemorrhagic disorder in clinical practice [19]. While strides have been made in understanding its pathophysiology and developing therapeutic interventions, leading to improved initial response rates [20], a substantial proportion of patients—nearly half—remain afflicted by persistent treatment failure or rapid relapse. This drives the disease into a chronic or refractory state [21,22]. Therefore, conducting a more in-depth exploration of the pathogenesis of ITP and finding new ITP-specific therapeutic methods remain the hot and difficult issues to be resolved in this field.

The etiology of ITP in bone marrow cell morphology is due to the maturation disorder of platelet-producing megakaryocytes, which is closely related to the differentiation cycle of megakaryocytes, cell death, and apoptosis [23,24]. Therefore, the study of cell cycle-related mechanisms is a key focus in ITP research. We have identified precursors of various immune cells and found a decreasing trend of pre-B cells in the bone marrow of ITP patients, consistent with previous reports of a reduction in total B cells in ITP bone marrow [18]. Pre-B cells exhibit significant immune dysregulation in ITP, which aligns with previous research findings [18]. Pre-B cells may contribute to the defect in megakaryocyte production present in ITP [25]. Although previous studies have reported the heterogeneity of mature megakaryocytic progenitor cells and pre-B cells in the bone marrow of ITP patients as potential targets for ITP treatment, our study, for the first time, revealed the specific roles of EBMP cells and pre-B1 cells in the pathogenesis of ITP through single-cell sequencing analysis. Our research not only confirmed the expansion of EBMP cell numbers and the significant depletion of pre-B1 cells in ITP but also, crucially, identified the core molecular mechanisms responsible for these phenotypic shifts. Specifically, we found that dysregulation of cell cycle- and cell death-related pathways represents the dominant driving force. The pathogenicity of ITP stems from functional abnormalities in core bone marrow subsets. For pre-B1 cells, our data reveal a series of abnormally expressed genes enriched in the pathways of programmed cell death. Mechanistically, the enrichment of cell cycle-related pathways suggests that pre-B1 cells may undergo aberrant cell cycle arrest in ITP. This proliferative arrest subsequently triggers or accelerates the apoptotic process, ultimately leading to the significant reduction in the pre-B1 cell population. In contrast, while pathways such as phagosome function or EBV infection were enriched in our analysis, they primarily reflect secondary inflammatory responses or accompanying changes rather than the primary drivers of the abnormal cell proportions. Therefore, we emphasize that the dysregulation of cell cycle progression and apoptotic signaling in pre-B1 cells is the key molecular basis underlying the characteristic depletion of this subset. For EBMP cells, the upregulation of key genes such as CXCL8 and IRF1, coupled with the activation of TNF and apoptosis pathways, indicates functional maturation defects and immune dysregulation. These changes, driven by aberrant death and signaling pathways, contribute to the expansion of EBMP cells and their pathogenic interaction with the hematopoietic microenvironment in ITP. Collectively, our findings pinpoint cell cycle and cell death as the central mechanisms regulating the fate of both pre-B1 and EBMP cells, providing novel insights and potential therapeutic targets for ITP.

Another finding of this study is the increase in the EBMP cell population in the ITP group, which was confirmed by our own immunofluorescence staining results from single-cell sequencing. Notably, among the significantly differential genes obtained by comparing the ITP group with the control group, the TNF signaling pathway, the intestinal immune network for IgA production, protein processing in the endoplasmic reticulum, and apoptosis-related KEGG pathways were significantly enriched. These results suggest functional changes in the EBMP cell population, which may also be an important mechanism and potential target for EBMP involvement in ITP progression [26]. Additionally, the abnormality in immune-related pathways of EBMP may also be a key process in ITP, especially in immune responses, responses to cytokines, leukocyte differentiation, regulation of cytokine production, and regulation of immune system processes [27]. At the gene level, the upregulation of *CXCL8* and *IRF1* in EBMPs may be key genes in regulating the functional abnormalities of EBMPs and the progression of ITP [28]. Conversely, targeting these genes may have potential clinical application value for the treatment of ITP.

The upregulation of CXCL8 and IRF1 in the EBMP cells of ITP patients is particularly noteworthy. CXCL8, a potent chemoattractant for neutrophils, is known to be involved in the recruitment of immune cells to sites of inflammation. Its increased expression in ITP patients could potentially enhance immune cell infiltration and activation, exacerbating the autoimmune response. IRF1, a transcription factor critical for the regulation of type I interferon and other immune response genes, is also upregulated in ITP. This finding suggests a possible role of IRF1 in the immune dysregulation observed in ITP. The high expression of CXCL8 and IRF1, together with the enrichment of immune-related pathways, indicates a proinflammatory environment in ITP, which may contribute to the activation of autoreactive immune cells and subsequent platelet destruction. Modulating the expression or activity of CXCL8 and IRF1 may help restore immune balance and alleviate autoimmune damage to platelets. Contrary to our initial hypothesis, further correlation analysis showed no significant association between CXCL8 expression levels and the proportion of EBMP cells in ITP samples (Figure 5G, *p* > 0.05). Future studies are warranted to validate the therapeutic potential of targeting these molecules in preclinical models of ITP.

The pathogenesis of ITP involves not only the functional abnormalities of individual cell populations, such as the dysfunction of megakaryocytes leading to impaired platelet production, which may be one of the mechanisms of ITP [29], but also disordered intercellular communication, which serves as a key driver in ITP progression. In this study, intercellular communication analysis identified a set of ligand–receptor pairs with diminished crosstalk between EBMP cells and MkP1 cells in ITP, among which LGALS9-CD44 was the most prominent [30]. The interaction signal of this pair was significantly higher in the control group than in the ITP group, which was further validated in our clinical samples. These findings indicate that the weakened LGALS9-CD44 interaction between EBMP cells and MkP1 cells may contribute to ITP pathogenesis, and restoring this signaling axis may represent a potential therapeutic strategy for ITP.

LGALS9 is an immunoregulatory protein that binds multiple receptors including CD44 [31]. Accumulating evidence has demonstrated that LGALS9–CD44 interaction modulates immune homeostasis, especially by regulating the stability and function of regulatory T cells [32,33]. In ITP, LGALS9 levels have been associated with disease activity, and CD44 has been widely implicated in platelet production and immune regulation. Our single-cell data further revealed markedly differential expression of LGALS9 and CD44 between EBMP cells and MkP1 cells, supporting the notion that this axis may be a key pathway involved in ITP progression. Notably, immunofluorescence co-localization analysis confirmed the spatial overlap of LGALS9 and CD44 proteins in the bone marrow microenvironment. However, this technique cannot definitively distinguish between intercellular ligand–receptor interactions and intracellular co-expression; thus, we interpret this observation as a co-localization phenomenon rather than direct cell–cell binding.

In the context of thrombocytopenia, the LGALS9-CD44 interaction has been reported to affect platelet development and function [34]. Platelets are derived from bone marrow megakaryocytes, and their production and activation are tightly controlled by multiple cytokines and signaling pathways [35,36]. Mechanistically, LGALS9 may regulate platelet formation and survival by binding to CD44 on megakaryocytes or platelets [37]. In addition, this interaction may modulate the immunoregulatory roles of platelets by influencing their crosstalk with leukocytes, thereby participating in inflammatory and immune responses [38]. However, the precise molecular mechanisms by which the LGALS9-CD44 axis affects megakaryocyte development and platelet production in ITP remain to be further elucidated.

Nevertheless, this study has several critical limitations. Its single-center design and limited sample size constrain the generalizability of the findings, which might be validated in larger, multi-center cohorts. The research relies on sequencing and histological validation, lacking functional in vitro or in vivo experiments such as gene editing or animal models. Consequently, the mechanistic insights remain preliminary and correlative rather than causative. In addition, our study focused exclusively on the bone marrow microenvironment and did not systematically obtain matched peripheral blood B cell data from the same individuals, which prevented us from analyzing the correlation between circulating B cell levels and bone marrow pre-B cell populations. These limitations reduce the representativeness of the results and preclude confirmation of a causal relationship between EBMP cell abnormalities, LGALS9-CD44 axis defects, and ITP pathogenesis. Therefore, the observed EBMP expansion and LGALS9-CD44 axis impairment represent preliminary hypotheses, not definitive pathogenic evidence. Any therapeutic implications must be interpreted with caution, and the identified molecular targets are exploratory, requiring further investigation before clinical translation.

In conclusion, single-cell transcriptome sequencing and immunofluorescence analysis of in-house samples revealed a significant increase in EBMP cells and a decrease in pre-B1 cells in the ITP group. We therefore focused on the functional and differential gene changes within these two cell populations in ITP. The upregulation of CXCL8, CSF1, and ITGB2 in EBMP cells may represent key regulators of EBMP dysfunction and ITP progression. From a cell–cell communication perspective, we further identified the loss of the LGALS9-CD44 interaction between EBMP and MkP1 cells as a potential contributor to ITP pathogenesis. Consequently, restoring the LGALS9-CD44 axis may offer a therapeutic strategy for improving ITP.

## 4. Materials and Methods

### 4.1. Clinical Sample Collection

Bone marrow samples were obtained from 15 ITP patients and 5 healthy donors. The clinical information of all 15 ITP patients and 5 healthy control individuals can be found in Table 1. This study was approved by the Medical Ethical Committee of the Seventh Affiliated Hospital of Sun Yat-sen University. The Ethics Committee’s ethical review approval number is KY-2024-022-02. This study was conducted in accordance with the Declaration of Helsinki.

### 4.2. Data Acquisition

The ITP and HC scRNA-seq data GSE196676 were accessed from the GEO database (https://www.ncbi.nlm.nih.gov/geo/query/acc.cgi?acc=GSE196676 (accessed on 30 March 2026)), which includes the CD34 + HSPCs of bone marrow (BM) samples of 4 patients newly diagnosed with ITP with no previous record of ITP treatment and 4 healthy controls. Then, we used the NormalizeData function in the Seurat package to normalize the expression matrix of each filtered sample.

### 4.3. Cell Count Verification

After obtaining the bone marrow samples from the ITP patients and healthy donors, we ensured that the initial quantities of cells were consistent between groups before they were processed for single-cell sequencing. The cell suspensions were counted using a hemocytometer, and equal numbers of cells were subjected to single-cell sequencing to ensure comparability. All 56,312 cells were acquired, including 27,805 cells in the HC group and 28,507 cells in the ITP group.

### 4.4. Quality Control of Cells and Principal Component Analysis

Firstly, we used the R package DropletUtils (version 1.30.0) to detect the expression of each cell and filter out barcodes that do not express any cells. Then, we further filtered based on the number of unique molecular identifiers (UMIs) in each cell. Next, we used the scater package to analyze the gene expression in cells and finally perform gene-level statistics.

After linear scaling of the data using the ScaleData function in the Seurat package, linear dimensionality reduction analysis (PCA) was performed using the RunPCA function in the Seurat package, followed by batch correction using the RunHarmony (version 1.2.4) function in the harmony package to eliminate differences between samples.

### 4.5. Dimensionality Reduction and Clustering

Firstly, we selected principal components (PCs) with high variance and conducted cell clustering analysis using the FindNeighbors and FindClusters functions in the Seurat package. Then, we performed non-linear dimensionality reduction analysis (UMAP) using the RunUMAP function in the Seurat package. We used the FindMarkers function in the Seurat package to calculate the differentially expressed genes between each cluster and all other cells (log2FC greater than or equal to 0.1, min cell group expression ratio of 0.25, *p*-value less than or equal to 0.05), thus obtaining marker genes (top 500 based on logFC). Subsequently, we annotated the cells based on these marker genes and visualized the clustering results. The FindMarkers function uses the parameter min.pct = 0.25 to filter out genes detected in less than 25% of cells, and logfc.threshold = 0.1 represents the minimum log2 fold change in average gene expression within the cluster compared to the average expression in all other cluster combinations.

### 4.6. Differential Expression Analysis

Differential expression analysis between the ITP and HC groups was performed using FindMarkers in the Seurat package for the core cell populations (pre-B1 and EBMP). The screening criteria were |fold change| > 1.2 and *p* value < 0.05.

### 4.7. Functional Enrichment Analysis

To investigate the potential functions of differentially expressed genes, we combined GO (Gene Ontology), KEGG (Kyoto Encyclopedia of Genes and Genomes), as well as the “clusterProfiler” package (version 4.16.0), where pathways with *p*-adjusted values less than 0.05 were considered significantly enriched. The GO annotations primarily cover three areas: cellular components (CC), molecular function (MF), and biological process (BP). Based on these three modules, the genes’ functions can be described from multiple dimensions.

### 4.8. Transcriptional Regulatory Network Analysis

We used SCENIC (version 1.3.1) to infer the regulatory activity of transcription factors corresponding to the respective data [39].

### 4.9. Analysis of Cell Interaction Network

Here, we used CellChat v1.1 to infer cell–cell communication based on the expression values of receptor–ligand genes corresponding to different cell types. Subsequently, we obtained the network relationships of receptor–ligand pairs between cells.

### 4.10. Double Immunofluorescence Staining Validation

Bone marrow biopsy specimens were fixed with 4% paraformaldehyde and mounted on glass slides. The slices were washed in PBS for 15 min to remove the OCT compound and boiled in antigen retrieval solution for 20 min. After natural cooling-off, the slides were incubated with 3% BSA for 1 h then incubated with CLC antibody (1:100 dilution, ThermoFisher, Waltham, MA, USA, Cat No: MA5-24251), CXCL8 antibody (1:100 dilution, Proteintech, San Diego, CA, USA, Cat No: 17038-1-AP), IRF1 antibody (1:100 dilution, Proteintech, Cat No: 11335-1-AP), CD41 antibody (1:100 dilution, Proteintech, Cat No: 24552-1-AP), CD44 antibody (1:100, Proteintech, Cat No: 60224-1-lg), and LGALS9 (1:100 dilution, Proteintech, Cat No: 17938-1-AP) at 4 °C overnight. The slices were washed in PBS three times and incubated with CoraLite594-conjugated Goat Anti-Rabbit IgG (H + L) (1:200, Proteintech, Cat No:SA00013-4), or CoraLite488-conjugated Goat Anti-Mouse IgG (H + L) for 1 h at room temperature. Finally, we washed the slices in PBS three times and stained the nucleus with DAPI for 10 min. All the samples were analyzed through confocal microscopy (Zeiss, Jena, Germany).

### 4.11. Statistical Analysis

The single-cell RNA-sequencing analysis was conducted with R software (Version 3.5). The data were analyzed with GraphPad Prism 8.0, which was also utilized for drawing the graphs. Various cell proportion inter-group differences were analyzed using the Wilcoxon rank-sum test; violin plots for inter-group comparisons were also analyzed using the Wilcoxon rank-sum test; specific gene differential expression tests were conducted using the Wilcoxon rank-sum test; and inter-group differential bar graph analysis for SCENIC was performed using the *t*-test. *p* values less than 0.05 indicated statistical significance.

## Figures and Tables

**Table 1:** Clinical characteristics of active ITP patients.

	ITP Patients	Healthy
Total (*n*)	15	5
Males (*n*)	7	2
Females (*n*)	8	3
Age (years (median, range))	39 (18, 67)	35 (19, 53)
Course of disease (days (median, range))	48 (2, 180)	/
Platelet counts	13 (2, 33)	218 (153, 306)
Newly diagnosis	15	/

Note: In this table, the symbol “/” indicates “not available (NA)”.

## Data Availability

The raw data supporting the conclusions of this article will be made available by the authors on request.

## Supplementary Materials

The following supporting information can be downloaded at https://www.mdpi.com/article/10.3390/ijms27083535/s1.
