# Supplementary material for: Single-Cell Analysis Highlights Pivotal Role of Eosinophil–Basophil Mast Cell Progenitor-Related Mechanism in Primary Immune Thrombocytopenia
Source: Int J Mol Sci. 2026 Apr 15;27(8):3535. doi: 10.3390/ijms27083535 (PMC13115725; doi:10.3390/ijms27083535)
Supplement: Supplementary file 1 [file ijms-27-03535-s001.zip › Supplementary Figure S1.pdf]

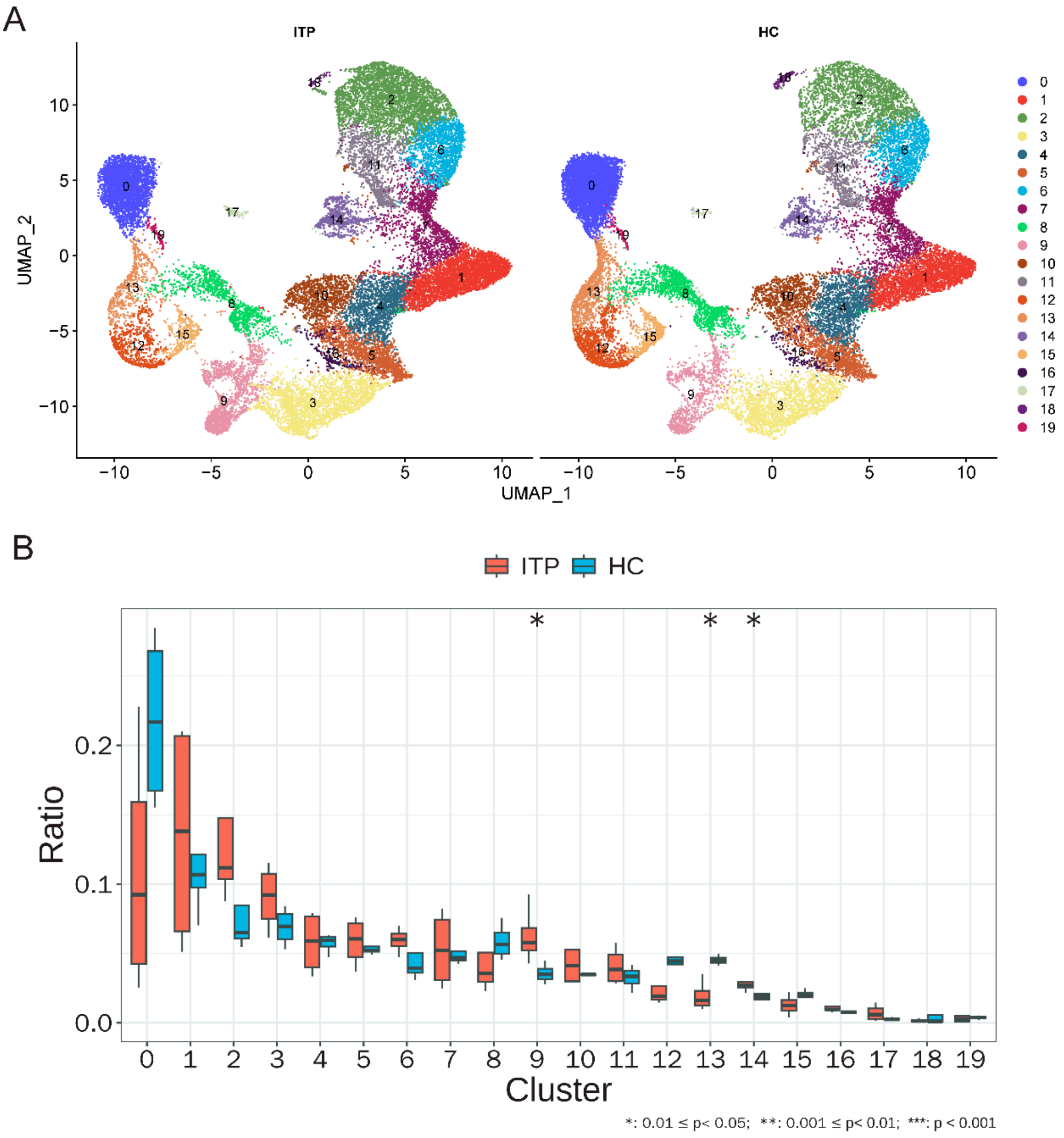

Supplementary Figure S1. Differential analysis of clusters in between ITP and HC groups.

(A)UMAP plot showing clusters of ITP and HC bone marrows.

(B)The bar plots show the ratio of per annotated clusters in TIP and HC groups.
