# Supplementary material for: Single-Cell Analysis Highlights Pivotal Role of Eosinophil–Basophil Mast Cell Progenitor-Related Mechanism in Primary Immune Thrombocytopenia
Source: Int J Mol Sci. 2026 Apr 15;27(8):3535. doi: 10.3390/ijms27083535 (PMC13115725; doi:10.3390/ijms27083535)
Supplement: Supplementary file 1 [file ijms-27-03535-s001.zip › Supplementary Figure S2.pdf]

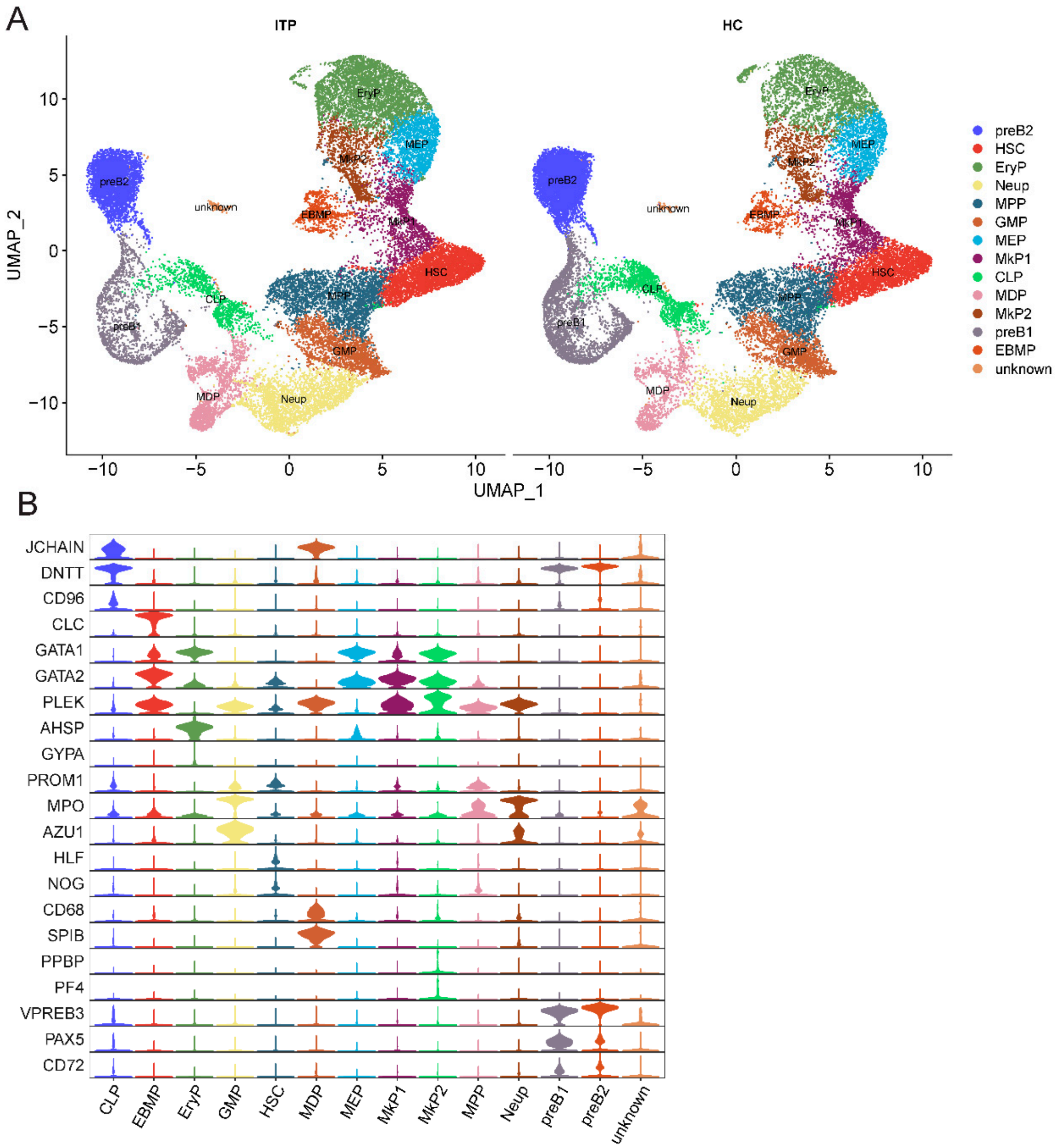

Supplementary Figure S2. Differential analysis of cell types in between ITP and HC groups. (A) Cell types were visualized in both ITP and HC groups using UMAP. Colors indicate clusters. (B) Violin plots showing the expression of specific marker genes in each cell types.
