# Supplementary material for: Single-Cell Analysis Highlights Pivotal Role of Eosinophil–Basophil Mast Cell Progenitor-Related Mechanism in Primary Immune Thrombocytopenia
Source: Int J Mol Sci. 2026 Apr 15;27(8):3535. doi: 10.3390/ijms27083535 (PMC13115725; doi:10.3390/ijms27083535)
Supplement: Supplementary file 1 [file ijms-27-03535-s001.zip › Supplementary Figure S3.pdf]

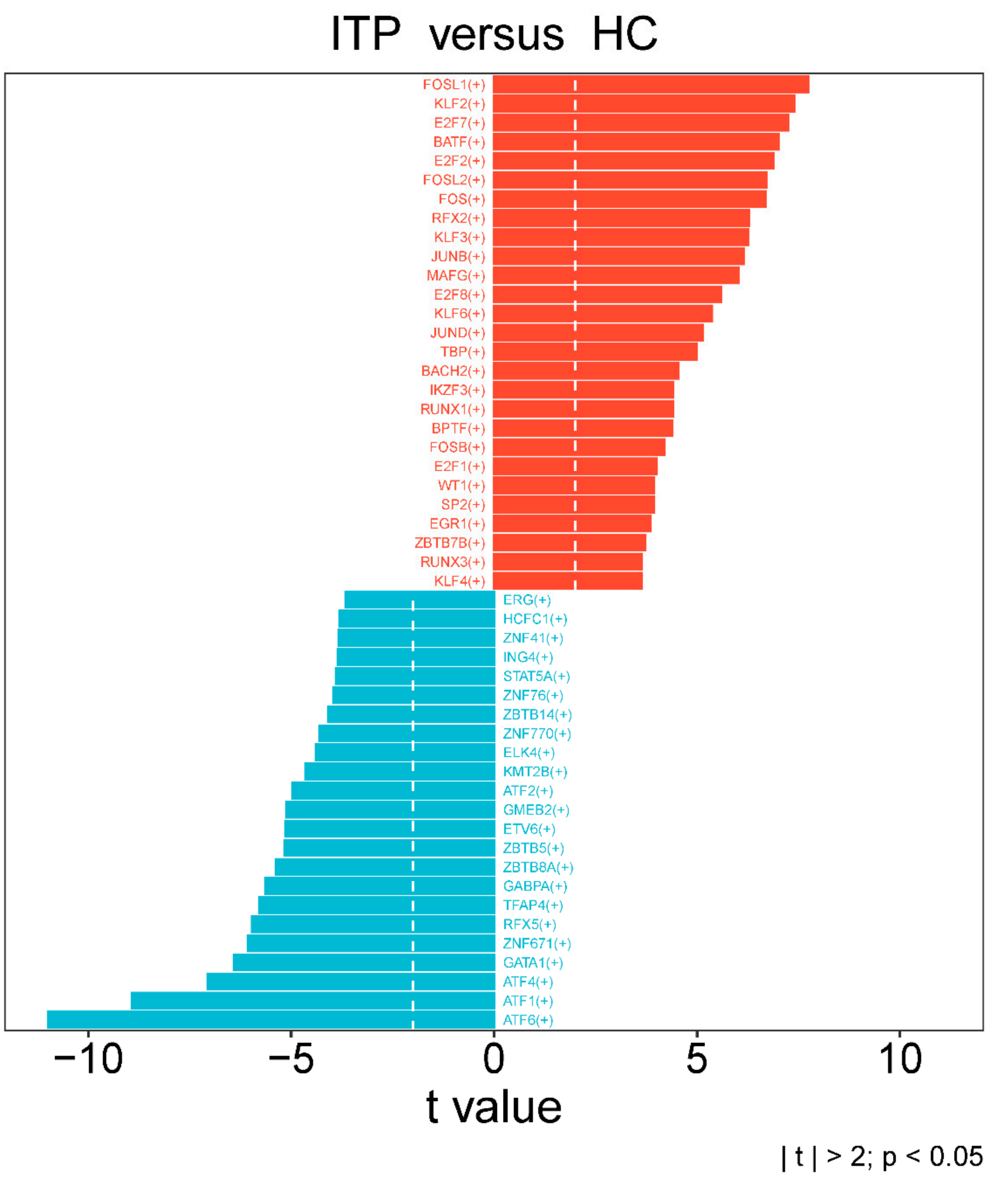

Supplementary Figure S3. SCENIC analysis indicated significant regulons for DEGs of pre-B1 cell in ITP.
