# Supplementary material for: Single-Cell Analysis Highlights Pivotal Role of Eosinophil–Basophil Mast Cell Progenitor-Related Mechanism in Primary Immune Thrombocytopenia
Source: Int J Mol Sci. 2026 Apr 15;27(8):3535. doi: 10.3390/ijms27083535 (PMC13115725; doi:10.3390/ijms27083535)
Supplement: Supplementary file 1 [file ijms-27-03535-s001.zip › Supplementary Figure S4.pdf]

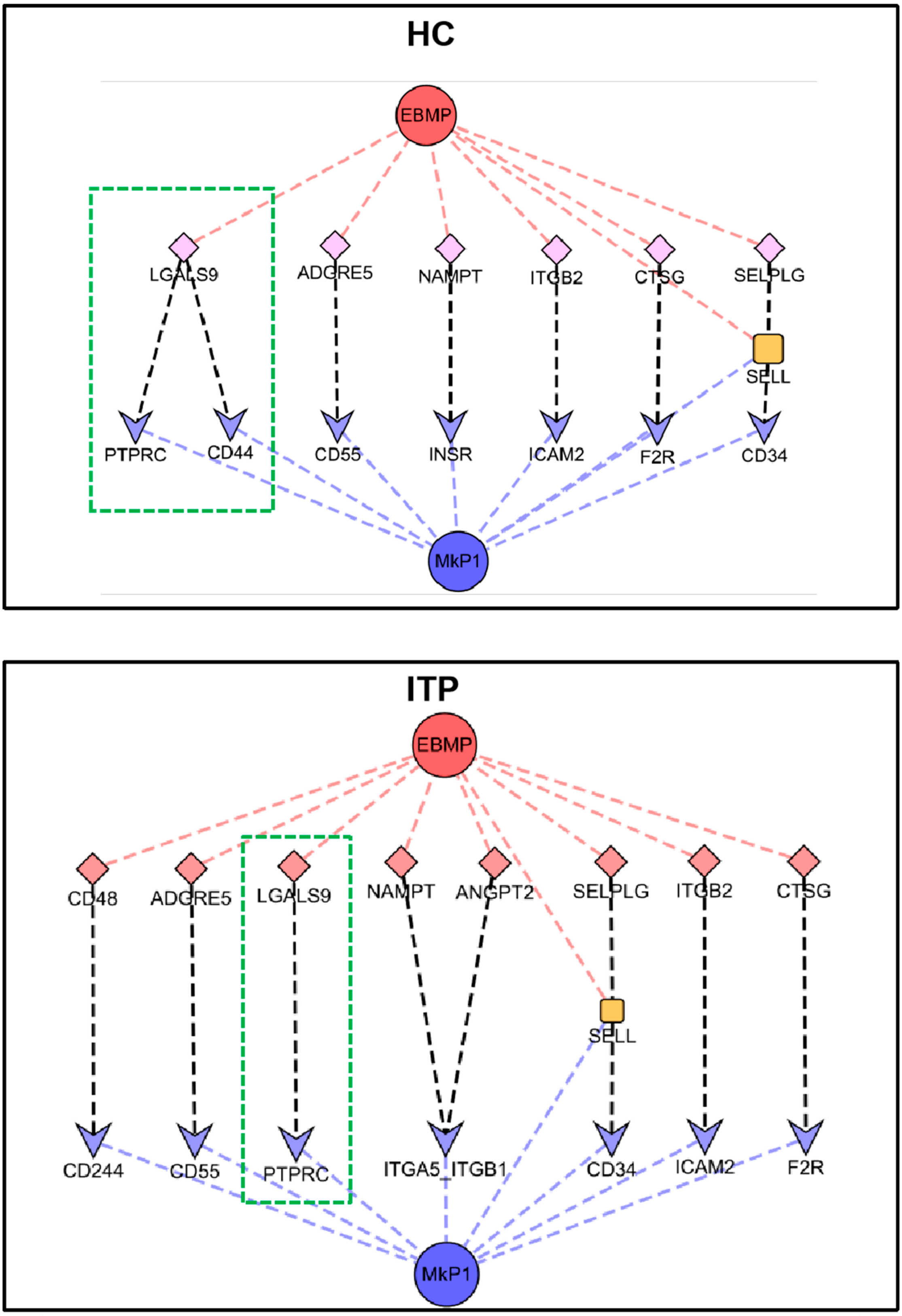

Supplementary Figure S4. The ligands on the EBMP cells of HC and ITP interact with the receptors on the MkP1 cells.
